# Supplementary figures and images for: Determination of the Mechanisms of MCPA Resistance in Amaranthus powellii
Source: Plant Direct. 2025 Sep 11;9(9):e70105. doi: 10.1002/pld3.70105 (PMC12426415; doi:10.1002/pld3.70105)

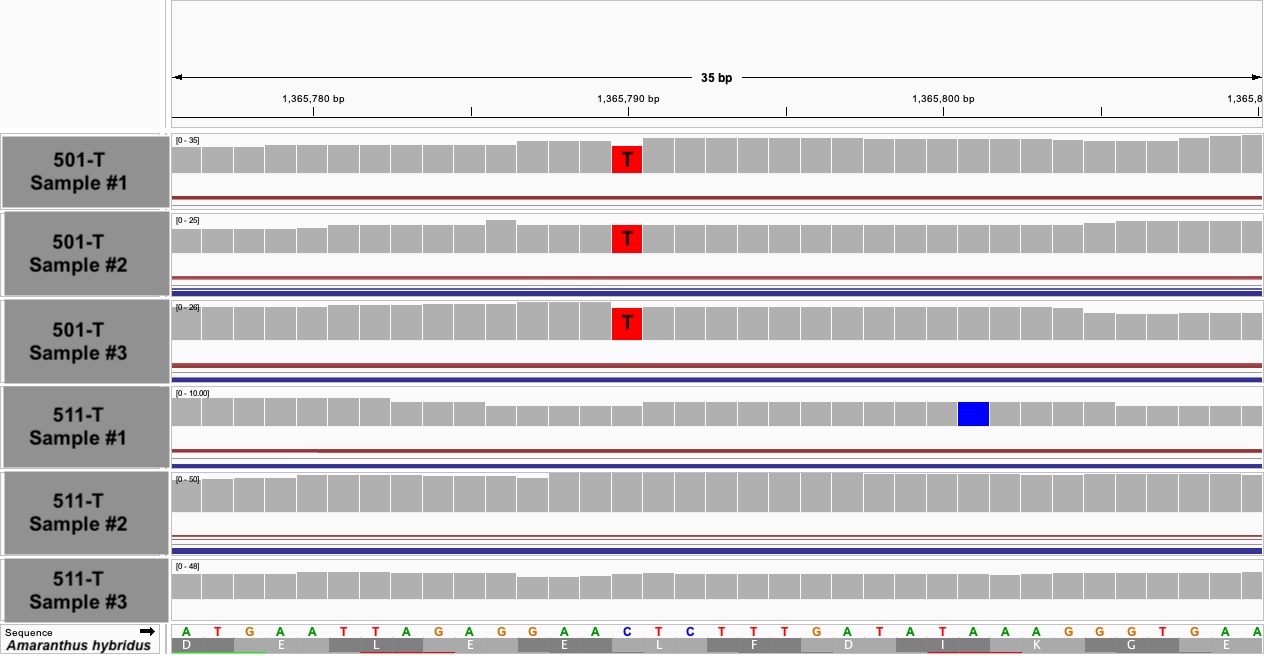

Supplement: Supplementary file 2 — Figure S1: Comparison of auxin response factor 9 (ARF9) sequence data between three untreated individuals from population 501 (MCPA‐resistant) and three untreated individuals from population 511 (MCPA‐susceptible) when aligned to the Amaranthus hybridus genome. The figure demonstrates a single nucleotide polymorphism conferring a leucine (Leu) to phenylalanine (Phe) substitution at this location in ARF9. [file PLD3-9-e70105-s001.jpg]

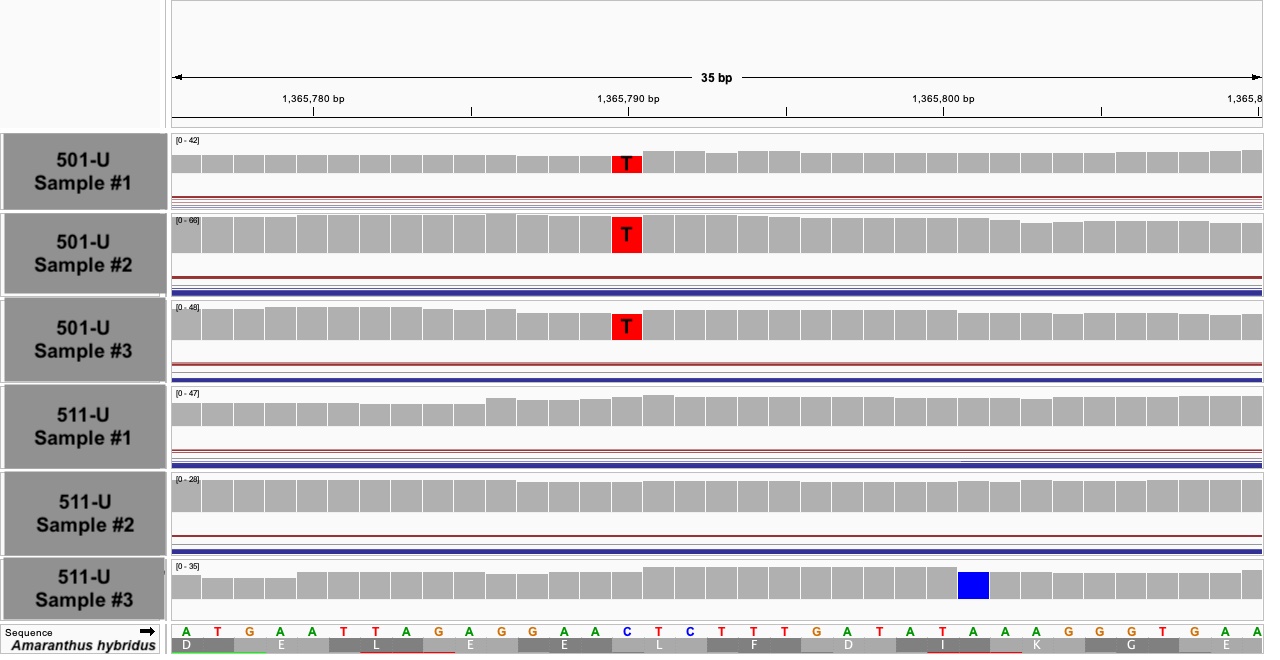

Supplement: Supplementary file 3 — Figure S2: Comparison of auxin response factor 9 (ARF9) sequence data between three treated individuals from population 501 (MCPA‐resistant) and three treated individuals from population 511 (MCPA‐susceptible) when aligned to the Amaranthus hybridus genome. The figure demonstrates a single nucleotide polymorphism conferring a leucine (Leu) to phenylalanine (Phe) substitution at this location in ARF9. [file PLD3-9-e70105-s002.jpg]
