# Supplementary material for: Determination of the Mechanisms of MCPA Resistance in Amaranthus powellii
Source: Plant Direct. 2025 Sep 11;9(9):e70105. doi: 10.1002/pld3.70105 (PMC12426415; doi:10.1002/pld3.70105)
Supplement: Supplementary file 1 — Data S1: Peer Review. [file PLD3-9-e70105-s003.pdf]

Plant Direct
Open Access

Determination of the mechanisms of MCPA resistance in Amaranthus powellii

Dr. Isabelle Aicklen, Dr. Mithila Jugulam , Dr. Todd A. Gaines , William Kramer , Martin Laforest ,
Darren Robinson , Peter Sikkema , Fran�ois Tardif

Decision Letter Round 1:

May 9, 2025

Dr. Isabelle Aicklen

University of Guelph

Guelph

Canada

RE: Determination of the mechanisms of MCPA resistance in Amaranthus powellii

Dear Dr. Aicklen:

Thank you for submitting to Plant Direct. All required reviews have been returned and we have now finished our evaluation of your manuscript. In light of the reviewers' and editor's comments, further revisions are needed before the paper can be accepted for publication in Plant Direct.

Please view the editors' and reviewers' comments below and use their suggestions as a guide while you work on your revision.

When uploading the revised version of this article, please be sure to include the following:

-A word document that contains your response to the reviewers. You should respond to each reviewer comment and note the changes made to the manuscript. If you do not agree with a reviewer's comment and choose not to make a suggested revision, please explain why. Please try to provide as complete an answer as possible to each reviewer's criticisms in the "response to reviewers" section.

-A tracked changes document

- A clean version of the latest version of the manuscript

First, let me apologize for the slow processing of this manuscript. It has been difficult to find reviewers. In the interest of time, I decided to reach a decision with only one review. The reviewer rises important issues that need to be addressed. Primarily, the approach to identify a putative gene responsible for the resistance trait is biased, and thus the conclusion is not justified. While herbicide resistance is not my area of expertise, I agree that a more non-biased approach should be attempted for candidate gene identification through SNP analysis. Please include a detailed response to the

reviewer comments, including changes made and any rebuttal, if you decide to submit a revised version.

To upload your revision, please click the link below.

https://plantdirect.msubmit.net/cgi-bin/main.plex?el=A4Lr1mf6A6pHe2I4A9ftdx19vE0cTFs2clsPd4BJuwZ

In order to provide as timely a service as possible, we ask that your revision is resubmitted within three months after receipt of this request. If an extension is needed, please send a request, along with a brief explanation, to the editorial office at plantdirect@wiley.com .

Thank you very much for giving us an opportunity to review your work. I look forward to receiving the next version.

Sincerely,

Gustavo MacIntosh

-- Editor comments:

First, let me apologize for the slow processing of this manuscript. It has been difficult to find reviewers. In the interest of time, I decided to reach a decision with only one review. The reviewer rises important issues that need to be addressed. Primarily, the approach to identify a putative gene responsible for the resistance trait is biased, and thus the conclusion is not justified. While herbicide resistance is not my area of expertise, I agree that a more non-biased approach should be attempted for candidate gene identification through SNP analysis. Please include a detailed response to the reviewer comments, including changes made and any rebuttal, if you decide to submit a revised version.

-- Reviewer comments:

Reviewer #1:

General Summary

Researchers thoroughly investigated potential mechanisms of MCPA resistance in a Amarnathus powellii and concluded that it is most likely TSR. They propose a single mutation in ARF9 as a potential marker/causal agent of R.

General Comments

The paper is exceptionally well written. While the authors primarily present negative results, it narrows down the potential sources of resistance and leads to the justified hypothesis that it is most likely TSR.

My biggest issue with the paper is how they performed their SNP analysis. I believe a more systematic (less human-biased) approach could have been taken where first all the potential resistance-causing SNPs were identified (i.e. all non-synonymous mutations). From their this master list could have been subset into known auxin related genes and then further refined to candidate genes. The authors may still only find this one snp as interesting, but the methodology would be

clearer. This may even be the methods the authors used but methods for SNP ID were not included in the methods section.

My other criticism is finding a SNP that differentiates two populations, even a non-synonymous mutation, is not uncommon. There are most likely hundreds of such SNPs. Without careful consideration of the germplasm (i.e. genomic background homogenization) or a crossing scheme that links the SNP to the phenotype, the claim that this SNP causes resistance is barely justifiable and is, at best, a very preliminary hypothesis.

Line Comments

Ln55: I am not sure I like the phrasing "amplifying the function of auxins". It makes it sound as if SAHs are somehow interacting with/amplifying the effect of natural auxin. I think it is more appropriate to say they are acting in competition with natural auxins and are outcompeting them - their by mis-regulating cell division and elongation, trophic responses, and senescence or something similar.

Ln77: It may be worthwhile to have a short discussion on why SAHs are selective for dicots. The fact that most of this selectivity is due to metabolism/NTSR would support the hypothesis that herbicide metabolism genes would be common for weed resistance and better justify starting there with your experiments. I.e. make the fact that you found TSR more exciting.

Ln 125: Your discussion of resistance to SAHs in Amaranthus is good. One thing that could be elaborated on is if there is any MCPA resistance in any other weed species, and if those mechanisms are known. If not, and this is the first case of a mechanism of resistance to MCPA, that greatly heightens the novelty and impact of this work. Make it explicit whether this is the only species that has demonstrated MCPA resistance.

Ln 259: You should have the version numbers for any programs you used or at least when the program was installed for reproducibility. This goes for STAR and all other programs mentioned in this methods. You have it for most programs just not all.

Ln 322: Did you not do a high-throughput NSP calling pipeline to identify all SNPs that may cause resistance? Maybe something like SNPeff? Then you could filter all non-synonymous mutations (i.e. protein changing SNPs), and then further filer by auxin-related genes for a more systematic approach rather than cherry picking SNPs by eye...if the later is what you did that should be made clear in the methodology as well. How did you chose the Auxin-related genes you looked at to find your SNP of interest?

Ln362-375: I am not sure this level of justification is needed, especially in the results. I think you could remove a lot of this from this section (and others) and save it more for the discussion. Leave the results a stricter reporting of the results.

Ln 387: Why is your snp calling methods only mentioned here and not your methods?

Ln390: Why were these genes targeted? Why not a lot more IAAs? Shouldn't there be many IAA genes in this analysis?

Ln400: This is a bit of a weak result. Finding a single nonsynonymous SNP between two populations, even if it is in a auxin-related gene, doesn't necessarily mean that it is causing the resistant phenotype. I would expect that between your two lines there would be hundreds, if not thousands of such snps. If there was a cross and this SNP co-segregated with your phenotype or some sort of population study where every MCPA-R population had this snp but no MCPA-S populations did, this would solidify this SNP as the resistance snp, or at least highly linked to resistance as a marker.

Ln 481: You are spending a lot of words and time discussing gene, in detail, that ultimately are not found to be involved in resistance. I suggest going through these genes faster to get to the interesting finding quicker. I find myself skimming this section looking for your positive result.

Ln 482: this is where it starts to get good and I think can stay as is.

Ln519: I think you need to remind the reader that you found this one mutation in a ARF9 and that due to the reasons you describe in 504-519, if may be involved in resistance. Just in case they do not read your results thoroughly, they should be reminded.

Figures: Why is there no nicely formatted alignment figure showing the SNP of interest. Isn't that the major conclusion of the paper? The supplemental figures do it, but they are ugly and hard to interpret. Also, what is the benefit of separating untreated from treated. At the DNA level there is no differences in the alignment and all six can be considered Reps for SNP analysis and visualization.

Decision Letter Round 2:

August 25, 2025
Dr. Isabelle Aicklen
University of Guelph
Guelph
Canada

MSID: 2025-01709R1
MS TITLE: Determination of the mechanisms of MCPA resistance in Amaranthus powellii

Dear Dr. Isabelle Aicklen:

I am pleased to inform you that your manuscript "Determination of the mechanisms of MCPA resistance in Amaranthus powellii" has been accepted for publication in Plant Direct.

Your article will appear online in the next available issue of Plant Direct. To ensure your article gets published as quickly as possible, please pay attention to the steps detailed below. We have found that most of the delays happen at this stage, especially at the payment stage, so please respond as quickly as possible when prompted.

License Agreement: Once your article has been accepted it will move to Production and undergo

admin and file checking - you may receive an email with any queries we have at this stage. When all required items are received by the publisher and queries resolved, the corresponding author will receive an email from Wiley's Author Services system which will ask them to log on at https://authorservices.wiley.com/bauthor and will present them with the appropriate license for completion. Your article cannot be published until both the signed license agreement and payment of the article fee have been received.

Payment of the Open Access Article Publication Fee: All articles published in Plant Direct are fully open access: immediately and freely available to read, download and share. Plant Direct charges a publication fee to cover the publication costs. The corresponding author for this manuscript should have already received a quote from the payments team (cs-openaccess@wiley.com) with the estimated article publication fee; please email cs-openaccess@wiley.com if this has not been received. The corresponding author should log on to the Wiley Author Services site, where the publication fee can be paid by credit card or an invoice. Pro Forma can also be requested. Payment of the publication charge must be received before the article will be published online.

Proofs: You will have the opportunity to look over your paper once more when you receive the author proofs for your article. The proofs will be with you in approximately two weeks. Please note that, in addition to publishing reviewer comments, the author's responses to review comments will also be published alongside the final version of the paper. If you would not like the author's responses to be published, please contact the editorial office at plantdirect@wiley.com .

Promotion of your article: You can help your research get the attention it deserves! Check out Wiley's free Promotion Guide for best-practice recommendations for promoting your work at www.wileyauthors.com/eeo/guide. And learn more about Wiley Editing Services which offers professional video, design, and writing services to create shareable video abstracts, infographics, conference posters, lay summaries, and research news stories for your research at www.wileyauthors.com/eeo/promotion.

Thank you again for your contribution to Plant Direct. If you have any questions, feel free to contact the editorial office at plantdirect@wiley.com .

Sincerely,
Gustavo MacIntosh
Gustavo MacIntosh
Editor, Plant Direct

Response Letter Round 1:

2025-01709 - Response to reviewers Comments from: Response/Action Taken Editor First, let me apologize for the slow processing of this manuscript. It has been difficult to find reviewers. In the interest of time, I decided to reach a decision with only one review. The reviewer rises important issues that need to be addressed. Primarily, the approach to identify a putative gene responsible for

the resistance trait is biased, and thus the conclusion is not justified. While herbicide resistance is not my area of expertise, I agree that a more non-biased approach should be attempted for candidate gene identification through SNP analysis. Please include a detailed response to the reviewer comments, including changes made and any rebuttal, if you decide to submit a revised version. Additional details have been added to the materials and methods section to address the methodology that was used for candidate gene identification and SNP analysis. Reviewer #1 The paper is exceptionally well written. While the authors primarily present negative results, it narrows down the potential sources of resistance and leads to the justified hypothesis that it is most likely TSR. My biggest issue with the paper is how they performed their SNP analysis. I believe a more systematic (less humanbiased) approach could have been taken where first all the potential resistance-causing SNPs were identified (i.e. all non-synonymous mutations). From their this master list could have been subset into known auxin related genes and then further refined to candidate genes. The authors may still only find this one snp as interesting, but the methodology would be clearer. This may even be the methods the authors used but methods for SNP ID were not included in the methods section. My other criticism is finding a SNP that differentiates two populations, even a non-synonymous mutation, is not uncommon. There are most likely hundreds of such SNPs. Without careful consideration of the germplasm (i.e. genomic background homogenization) or a crossing scheme that links the SNP to the phenotype, the claim that this SNP causes resistance is barely justifiable and is, at best, a very preliminary hypothesis. Additional details have been added to the materials and methods section to address the methodology that was used for candidate gene identification and SNP analysis. The conclusions highlight that the results rule out the involvement of any major NTSR mechanism and point to a potential TSR mechanism. Ln55: I am not sure I like the phrasing "amplifying the Modified as suggested. function of auxins". It makes it sound as if SAHs are somehow interacting with/amplifying the effect of natural auxin. I think it is more appropriate to say they are acting in competition with natural auxins and are outcompeting them - their by mis-regulating cell division and elongation, trophic responses, and senescence or something similar. Ln77: It may be worthwhile to have a short discussion on why SAHs are selective for dicots. The fact that most of this selectivity is due to metabolism/NTSR would support the hypothesis that herbicide metabolism genes would be common for weed resistance and better justify starting there with your experiments. I.e. make the fact that you found TSR more exciting. Additional details have been added. Ln 125: Your discussion of resistance to SAHs in Amaranthus is good. One thing that could be elaborated on is if there is any MCPA resistance in any other weed species, and if those mechanisms are known. If not, and this is the first case of a mechanism of resistance to MCPA, that greatly heightens the novelty and impact of this work. Make it explicit whether this is the only species that has demonstrated MCPA resistance. Additional details have been added. Ln 259: You should have the version numbers for any programs you used or at least when the program was installed for reproducibility. This goes for STAR and all other programs mentioned in this methods. You have it for most programs just not all. Additional details have been added. Ln 322: Did you not do a high-throughput NSP calling pipeline to identify all SNPs that may cause resistance? Maybe something like SNPeff? Then you could filter all non-synonymous mutations (i.e. protein changing SNPs), and then further filer by auxin-related genes for a more systematic approach rather than cherry picking SNPs by eye...if the later is what you did that should be made clear in the methodology as well. How did you chose the Auxinrelated genes you looked at to find your SNP of interest? Additional details have been added to describe the methodology that was used to identify candidates for resistance

mutations. Ln362-375: I am not sure this level of justification is needed, especially in the results. I think you could remove a As suggested by the reviewer, some of these details have been moved to the lot of this from this section (and others) and save it more for the discussion. Leave the results a stricter reporting of the results. discussion. Ln 387: Why is your snp calling methods only mentioned here and not your methods? Additional details have been added in the methodology section. Ln390: Why were these genes targeted? Why not a lot more IAAs? Shouldn't there be many IAA genes in this analysis? The authors investigated SNPs in 29 IAA genes. These genes were targeted as they are the 29 IAA genes found in Arabidopsis. Ln400: This is a bit of a weak result. Finding a single nonsynonymous SNP between two populations, even if it is in a auxin-related gene, doesn't necessarily mean that it is causing the resistant phenotype. I would expect that between your two lines there would be hundreds, if not thousands of such snps. If there was a cross and this SNP co-segregated with your phenotype or some sort of population study where every MCPA-R population had this snp but no MCPA-S populations did, this would solidify this SNP as the resistance snp, or at least highly linked to resistance as a marker. The authors concluded that the resistance mechanism could not be linked to a NTSR mechanism and therefore is likely a SNP in the auxin pathway. As suggested by the reviewer, the identification of a SNP in ARF9 must be further validated through genetic crosses. Due to time constraints and technical challenges associated with making crosses between Amaranthus powellii populations, crosses could not be completed and so the authors are suggesting a preliminary hypothesis that indicates a TSR mechanism is the likely cause of SAH resistance in this population. Ln 481: You are spending a lot of words and time discussing gene, in detail, that ultimately are not found to be involved in resistance. I suggest going through these genes faster to get to the interesting finding quicker. I find myself skimming this section looking for your positive result. Modified as suggested. Ln 482: this is where it starts to get good and I think can stay as is. No changes made. Ln519: I think you need to remind the reader that you found this one mutation in a ARF9 and that due to the reasons you describe in 504-519, if may be involved in resistance. Just in case they do not read your results thoroughly, they Additional details added. should be reminded. Figures: Why is there no nicely formatted alignment figure showing the SNP of interest. Isn't that the major conclusion of the paper? The supplemental figures do it, but they are ugly and hard to interpret. Also, what is the benefit of separating untreated from treated. At the DNA level there is no differences in the alignment and all six can be considered Reps for SNP analysis and visualization. No changes made. The authors have chosen to keep these diagrams in their current state as they feel this accurately depicts the presence of a SNP in ARF9 in all resistant individuals that were sampled.
